# The effect of linkage disequilibrium on linkage analysis of incomplete pedigrees

**DOI:** 10.1186/1471-2156-6-S1-S6

**Published:** 2005-12-30

**Authors:** Douglas F Levinson, Peter Holmans

**Affiliations:** 1 Department of Psychiatry, University of Pennsylvania School of Medicine, 353 Market Street, Philadelphia, PA, USA; 2 Department of Psychological Medicine, School of Medicine, Cardiff University, Cardiff, UK

## Abstract

Dense SNP maps can be highly informative for linkage studies. But when parental genotypes are missing, multipoint linkage scores can be inflated in regions with substantial marker-marker linkage disequilibrium (LD). Such regions were observed in the Affymetrix SNP genotypes for the Genetic Analysis Workshop 14 (GAW14) Collaborative Study on the Genetics of Alcoholism (COGA) dataset, providing an opportunity to test a novel simulation strategy for studying this problem. First, an inheritance vector (with or without linkage present) is simulated for each replicate, i.e., locations of recombinations and transmission of parental chromosomes are determined for each meiosis. Then, two sets of founder haplotypes are superimposed onto the inheritance vector: one set that is inferred from the actual data and which contains the pattern of LD; and one set created by randomly selecting parental alleles based on the known allele frequencies, with no correlation (LD) between markers. Applying this strategy to a map of 176 SNPs (66 Mb of chromosome 7) for 100 replicates of 116 sibling pairs, significant inflation of multipoint linkage scores was observed in regions of high LD when parental genotypes were set to missing, with no linkage present. Similar inflation was observed in analyses of the COGA data for these affected sib pairs with parental genotypes set to missing, but not after reducing the marker map until *r*^2 ^between any pair of markers was ≤ 0.05. Additional simulation studies of affected sib pairs assuming uniform LD throughout a marker map demonstrated inflation of significance levels at *r*^2 ^values greater than 0.05. When genotypes are available only from two affected siblings in many families in a sample, trimming SNP maps to limit *r*^2 ^to 0–0.05 for all marker pairs will prevent inflation of linkage scores without sacrificing substantial linkage information. Simulation studies on the observed pedigree structures and map can also be used to determine the effect of LD on a particular study.

## Background

Linkage genome scans using dense maps of single nucleotide polymorphism (SNP) markers have been shown to provide greater information content than 10-cM microsatellite scans [1-5]. However, false positive peaks were observed in a SNP-based linkage study of prostate cancer in regions with marker-marker *D*' values greater than 0.6 [4]; and in simulations of pairs of markers with no linkage present, inflation of linkage scores was observed as marker-marker *D*' values increased between 0.4 and 0.8 [6]. The problem has not been systematically studied using the *r*^2 ^measure of linkage disequilibrium (LD).

The Collaborative Study on the Genetics of Alcoholism (COGA) datasets for Genetic Analysis Workshop 14 (GAW14) provided an opportunity to study this problem, because the Affymetrix SNP data revealed regions with high marker-marker LD. The main goal of the present analyses was to test a novel simulation strategy for studying the effect of LD on linkage scores with and without availability of parental genotypes.

## Methods

We selected the 116 European-ancestry pedigrees from the 143 families in the GAW14 COGA dataset. We focus here on analyses that used one pair of affected siblings per family plus parents, but additional analyses utilized the full pedigrees (485 affected and 287 unaffected genotyped individuals, including most parents); nuclear families (139 sibships, 473 affected and 127 unaffected genotyped individuals); or one sibship from each pedigree (390 affected and 264 unaffected individuals). In these additional analyses, comparisons of linkage results with different maps were similar to those reported in other GAW14 papers and so are not discussed in detail here; comparisons of information content for different maps, and analyses of the effects of LD on linkage scores, were consistent with those reported here for simulated data.

Genotypes were available for a 10-cM microsatellite map, 4,752 Illumina SNPs and 11,560 Affymetrix SNPs. In the 66 Mb of chromosome 7 containing the largest linkage peak in these 116 pedigrees (by multipoint analysis of microsatellite data), there were 212 Affymetrix SNPs. We excluded 36 of these because of deviation from Hardy-Weinberg equilibrium (*P *< 0.001), call rate < 0.8, or minor allele frequency < 0.05. We studied the remaining 176 SNPs ("High-LD map") and a subset of 109 SNPs ("Low-LD map") in which there was no pairwise *r*^2 ^value > 0.05.

Linkage analyses were carried out with ALLEGRO [7] (exponential model, S_pairs_, with families weighted to the power 0.5 of the variance of their expected scores without linkage). The *r*^2 ^LD statistic was computed with HAPLOVIEW [8], and correlation and regression statistics with SYSTAT 8.0.

To create founder haplotypes for simulation studies, we used ALLEGRO to infer 176-marker haplotypes for all individuals in the full pedigree data. ALLEGRO reports the most likely haplotype, with "0" alleles where no inference can be made. From inferred haplotypes with less than 5 "0" alleles, we selected 464 haplotypes from unrelated individuals, for use as parental haplotypes in the simulation study described below. Missing data were imputed based on COGA pedigree SNP allele frequencies. These haplotypes had the same LD pattern as the entire dataset (HAPLOVIEW).

Data were simulated using SIM (unpublished, A. Kirby) and programs written for this study. For each replicate, SIM assigned 2 unique alleles to each founder (e.g., founder 100 was assigned allele "199" for all markers on one chromosome and "200" on the paired chromosome), transmitted them to offspring by selecting locations of recombinations for each meiosis based on genetic distances, and then transmitted parental chromosomes (here, assuming no linkage, although the program can also follow a specified disease transmission model). Then, for each replicate two different datasets were created by replacing each unique allele with an allele from a corresponding founder haplotype (gene-dropping). First, we assigned to each parent 2 haplotypes from among the 464 haplotypes inferred from COGA data as described above (LD condition), and then we assigned parental haplotypes created by random selection of alleles based on the allele frequencies in the COGA data, with no correlation between markers (No-LD).

In addition, to examine the effects of varying levels of inter-marker LD more systematically, 5,000 replicates were created for 650 pedigrees containing 920 affected sib pairs affected sib pairs (ASPs) with 30% of parents genotyped, for 200 SNPs (0.2 cM apart) with no linkage present, with uniform LD at *r*^2 ^values between marker pairs of 0–0.4 in steps of 0.05.

## Results

**Figure 1:**
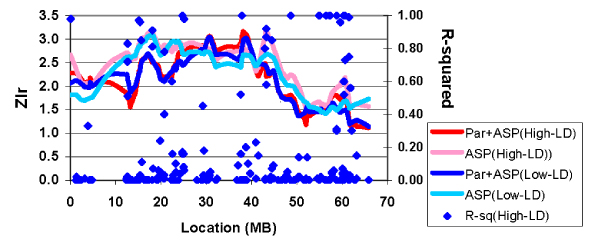
Linkage scores for 116 COGA ASPs: High vs. low LD maps with and without parental genotypes.

In analyses of the COGA dataset (pedigrees, sibships or ASPs), if parental genotypes were available, then there were minimal differences between linkage scores for the High-LD and Low-LD maps for the full pedigrees, sibships, or ASPs (data not shown). Figure 1 illustrates the analyses of 116 ASPs. Linkage scores (Zlr statistic) are shown for analyses either using the parental genotypes (Par+ASP) or setting them to missing (ASP), and using either the High-LD or Low-LD map. Also shown are *r*^2 ^values for each marker with the marker to its left, which gives a reasonable indication of the variation in LD throughout the region, although the pattern for all possible pairs is more complex. There are regions around 43 and 60 Mb where LD is substantial, and where Zlr is inflated when parental genotypes are not available for the High-LD map. Table 1 shows marker-marker *r*^2 ^values for the region around 60 Mb; values for all pairs of the 176 SNPs are available on request. In a third region, centered around 17–18 Mb, Zlr is inflated when parental genotypes are not available for both maps, suggesting that in this region the absence of parental data changes the scores.

**Figure 2:**
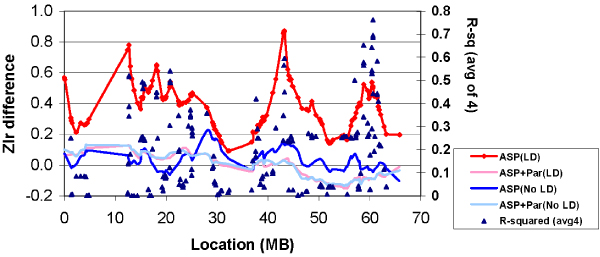
Mean difference between observed and "true" ZLr (no linkage).

Table 2 and Figure 2 show results for simulations of 100 replicates of 116 ASPs with no linkage present. First, linkage analysis was carried out for each replicate using the "true" inheritance vector (the recombination pattern and transmission of unique alleles created by SIM). Then, datasets created with LD and No-LD founder haplotypes were each analyzed with parental genotypes available (ASP+Par) or set to missing (ASP). For each dataset in each replicate, the Zlr score at each point from the analysis of the true inheritance vector was subtracted from the score for that dataset. Figure 2 shows the mean values of this subtraction at each point for each type of dataset. Table 2 shows Pearson correlations between the Zlr-difference at the location of each SNP and each of four variables: (a) pairwise *r*^2 ^between the SNP and the marker to its left; (b) distance (in Mb) between each pair of SNPs; (c) the average of 4 consecutive *r*^2 ^values (computed for every fourth SNP to avoid overlap) such that for SNPs with order 1–2–3–4–5, the Avg4-LD is the average of *r*^2 ^values for the pairs 1–2, 2–3, 3–4, and 4–5; and (d) the average of 4 consecutive distances, computed as described for Avg4-LD. The correlation coefficient was computed between the average Zlr difference (observed-true) at each marker position and either the *r*^2 ^or distance measure for that SNP. The best predictor (*r *= 0.52) of the observed-true difference for ASPs (LD map) without parental genotypes was the Avg4 *r*^2 ^measure. A multiple regression analysis was also computed, with Zlr difference for the ASP-LD dataset as the dependent variable, and Avg4-LD, Avg4-Distance and their interaction as independent variables. Only *r*^2 ^was predictive of Zlr difference (*P *= 0.0063). Note that in 19% of replicates with no linkage present, the maximum Zlr score between 41 and 45 Mb was > 2.0, and in 2% it was > 3.0.

**Figure 3:**
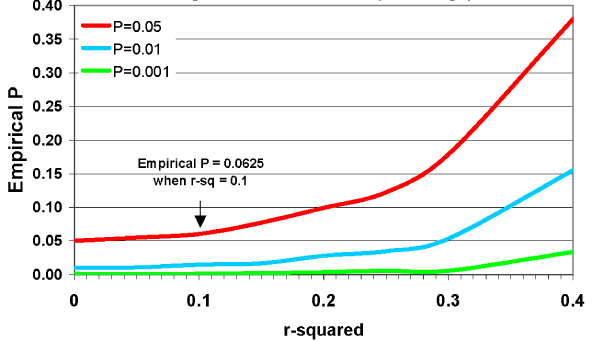
Effect of LD (*r*^2^) on significant thresholds (no linkage).

Finally, Figure 3 shows the results of simulations of maps of 200 markers, with no linkage to disease present, with uniform LD (*r*^2^) between each marker pair, for 650 pedigrees with 70% of parental genotypes. The red, blue and green lines show the proportion of replicates in which the largest linkage score exceeded the threshold observed 5, 1, or 0.1% of the time in the absence of LD (*r*^2 ^= 0). For each threshold, false positive results increase with *r*^2^. Even at *r*^2 ^= 0.10, the proportion of "significant" results was 0.0625 for the threshold associated with *P *= 0.05 for *r*^2 ^= 0.

## Discussion and Conclusions

These analyses support the conclusion that when parental genotypes are missing and cannot be reconstructed from the constellation of genotyped individuals, the presence of marker-marker LD can substantially inflate linkage scores [4,6]. In the real COGA data, this effect was clearly visible, although inconsistent. However, we only studied one small chromosomal region in the real dataset. In simulated data, where multiple replicates could be studied, the effect was highly significant. Thus, in genome-wide studies with many missing parental genotypes, one would expect that if strong LD was present in many regions, linkage scores would be inflated in some of them.

The data presented in Figure 3 suggest that when a dataset includes incomplete families, and especially ASPs without parents, an *r*^2 ^threshold of 0.05 is probably desirable. Fortunately, as shown in Figure 1, little linkage information is likely to be lost by using the densest map with all pairwise values of *r*^2 ^< 0.05. Alternatively, it might be possible to correct for LD statistically, although it may prove difficult to account for patterns of LD that extend beyond the adjacent two markers.

The simulation method described here can also be used to evaluate whether inflation of linkage scores is likely with a marker map and pedigree sample. One would first simulate replicates based on the pedigree structures in the real study as described above, with or without linkage present, assuming that all parental genotypes are available. Gene-dropping would then be carried out, using haplotypes inferred from the real data (and thus containing the observed LD pattern). After setting parental genotypes to missing, one would repeat the linkage analysis of each replicate for the "true" data (the unique alleles from the simulation) and the gene-dropping data that contain the LD pattern, compute the difference between these scores for each replicate, and determine whether the difference is correlated with *r*^2 ^values (such as the Avg4 measure described above).

## Abbreviations

ASP: Affected sib pair

COGA: Collaborative Study on the Genetics of Alcoholism

GAW: Genetic Analysis Workshop

LD: Linkage disequilibrium

SNP: Single nucleotide polymorphism

## Authors' contributions

DFL designed these studies and carried out the main analyses. PH participated in critical discussions of these ideas, reviewed the manuscript, and carried out the simulation study presented in Figure 3.

## Figures and Tables

**Table 1:** Marker-marker LD (*r*^2^) in one region (≈ 60 Mb).

SNP	Gap (bp)	157	158	159	160	161	162	163
157								
158	265	**0.60**						
159	3,337	**0.89**	**0.52**					
160	373	**0.90**	**0.51**	**1**				
161	211,379	0.07	0.06	0.05	0.05			
162	23	0.07	0.07	0.05	0.06	**1.00**		
163	1,504	0.07	0.06	0.05	0.05	**1.00**	**1.00**	
164	32,532	0.05	0.01	0.04	0.05	**0.72**	**0.73**	**0.73**

**Table 2:** Correlations between the difference between observed and "true" Zlr and measures of LD and distance (ASPs without parents).

**Correlation of Zlr difference (observed-true) with:**	**ASP(LD)**		**ASP(No LD)**	
		
	*r*	*P*	*r*	*P*
a) LD (pairwise *r*^2^, left)	0.37	0.0156	-0.12	0.4597
b) Distance (pairwise, left)	-0.30	0.0544	-0.09	0.5857
c) Avg4-LD (average of 4 consecutive *r*^2 ^values)	**0.52**	**0.0004**	-0.17	0.2874
d) Avg4-Distance (average of 4 consecutive distances)	-0.43	0.0044	0.16	0.2997
